# Pre-exposure to drought enhances resilience to recurrent drought in soybean by orchestrating H_2_O_2_ homeostasis through coordinated antioxidant and hexose metabolism

**DOI:** 10.3389/fpls.2026.1777878

**Published:** 2026-07-15

**Authors:** Wenqing Zhao, Yuqian Shen, Yuanyuan Qi, Lin Liu, Hanting Peng, Lei Li, Haihong Lai, Qingtao Gong, Haidong Jiang

**Affiliations:** Key Laboratory of Crop Physiology Ecology and Production Management, Ministry of Agriculture and Rural Affairs/College of Agriculture, Nanjing Agricultural University, Nanjing, China

**Keywords:** antioxidant oxidase, drought priming, drought stress, H_2_O_2_ homeostasis, soybean, sucrose metabolism, WRKY transcription factor

## Abstract

Drought-primed soybean seedlings are able to maintain a dynamic equilibrium of hydrogen peroxide (H_2_O_2_) during subsequent severe drought, but the regulatory mechanisms remain poorly understood. To investigate this, soybean seedlings in this study were exposed to mild drought priming (0.5% PEG6000 treatment for 3, 6, or 12h) followed by severe drought (5% PEG6000) for 24h. The results showed that a 6h drought-priming (W6S) effectively established dynamic H_2_O_2_ homeostasis under severe drought, maintaining levels 16.6%–19.6% above the control and minimizing oxidative damage within 0–24h. This response was associated with the dynamic regulation of WRKY transcription factors (TFs) (*GmWRKY33a/b* and *GmWRKY12*). Their early induction promoted peroxidase and catalase gene expression and activity, while subsequent downregulation maintained H_2_O_2_ balance. At 24h, reactivation of *GmWRKY33a/b* and *GmWRKY12* and enhanced sucrose cleavage into hexoses further supported homeostasis. In contrast, shorter (W3S) or longer (W12S) priming durations failed to induce this homeostasis and resulted in severe oxidative injury. Although lasting severe drought (12–24h) further elevated H_2_O_2_ levels, upregulated expression of *WRKY33 (a, b)* and *WRKY12* might increase soluble sugar levels to provide more substances and energy for maintaining H_2_O_2_ homeostasis and meeting the osmotic regulation. These findings demonstrate that optimal drought priming promotes H_2_O_2_ homeostasis through WRKY-mediated transcriptional regulation and hexose metabolism, providing potential targets for improving soybean drought resilience.

## 1 Introduction

Soybean (*Glycine max* (L.) Merrill), originating in China, is a major global crop for oil and protein production. Drought, as a critical abiotic stress, severely limits growth and yield by inducing oxidative stress and disrupting metabolic processes (Ahluwalia et al., 2021; Porcel and Ruiz-Lozano, 2004; Valliyodan and Nguyen, 2006). One key consequence of drought is the excessive accumulation of reactive oxygen species (ROS), particularly hydrogen peroxide (H_2_O_2_) (Castro et al., 2021). H_2_O_2_ functions as a central signaling molecule, regulating diverse physiological processes and defense responses against stress (Khan et al., 2018). As an early and ubiquitous signal, H_2_O_2_ interacts with multiple types of pathways, serving as a hub in cellular stress signaling networks (Mittler et al., 2022). Our previous studies demonstrated that mild drought priming enables soybean to establish H_2_O_2_ homeostasis under subsequent severe drought, thereby activating stress defense mechanisms. However, this balance cannot be achieved under prolonged mild or continuous severe drought (Shen et al., 2024; Tao et al., 2022). Thus, drought-primed plants exposed to recurrent or progressive drought may develop a distinct state of H_2_O_2_ homeostasis, though the underlying regulatory mechanisms remain poorly understood. It is well established that specific WRKY transcription factors (TFs), which regulate ROS metabolism, are rapidly and differentially expressed under drought stress (Jiang and Deyholos, 2009). For example, *ZmWRKY40* in maize reduces ROS levels in transgenic lines by enhancing catalase (CAT) and peroxidase dismutase (POD) activities, thereby improving drought tolerance (Wang et al., 2018a). Conversely, the overexpression of *WRKY10* leads to an increase in ROS accumulation in both chloroplasts and the apoplast, while concurrently activating genes associated with heat shock transcription factors and proteins (Chen et al., 2022). In tomatoes, *WRKY3* directly binds to the promoters of the *SlGRXS1* gene cluster, thereby promoting their expression and facilitating ROS scavenging (Wang et al., 2022). These findings suggest that WRKY proteins are integral components of ROS signaling pathways and play pivotal roles within the broader ROS signaling network. In soybeans, several studies have reported changes in WRKY expression in response to abiotic stress such as drought, salinity, and waterlogging. Notably, fluctuations in H_2_O_2_ content during stress have been associated with the differential expression of specific *WRKYs* in leaves (Song et al., 2016; Wang et al., 2015). Consequently, we hypothesize that alterations in *WRKY* expression under ‘progressive drought stress’ may also influence H_2_O_2_ homeostasis, thereby modulating drought resistance in soybean seedlings. However, research on this specific mechanism remains limited.

When plants undergo drought priming, additional energy is required to maintain H_2_O_2_ homeostasis during subsequent severe drought (Shen et al., 2024). Photosynthesis plays a central role in carbon fixation and metabolism by supplying energy for various physiological processes. Sucrose, the primary product of photosynthesis, is synthesized by sucrose phosphate synthase (SPS). A reduction in SPS activity limits sucrose production and decreases soluble sugar levels (Gutiérrez-Miceli et al., 2002). Drought stress adversely affects photosynthetic rates, disrupts carbon distribution and metabolism, and ultimately depletes energy reserves, leading to yield loss (Cuellar-Ortiz et al., 2008). Interestingly, drought conditions often enhance SPS activity, resulting in increased sucrose accumulation (Geigenberger et al., 1997; Xu et al., 2015; Yang et al., 2001). Subsequently, sucrose is broken down by sucrose synthase (SuSy) and invertase [both ac-id-INV (A-INV) and neutral-INV (N-INV)] into hexose in sink organs, thereby improving osmotic potential (Ruan, 2014). Importantly, WRKY TFs are closely linked to sugar metabolism, as they regulate carbohydrate biosynthesis and modulate sucrose synthesis. For instance, silencing *WRKY22* in grape increases SPS activity, enhances sucrose synthesis, and raises soluble sugar content, while *WRKY3* in Pitaya activates sucrose biosynthesis genes, promoting sugar accumulation (Wei et al., 2019). Although some studies have examined changes in sugar metabolism in soybean under varying levels of drought stress and after drought priming, the interactions among WRKY TFs, H_2_O_2_ homeostasis, and carbohydrate metabolism remain largely unexplored.

In this study, we utilized the drought-tolerant soybean cultivar Kefeng 1 and applied PEG6000 to simulate drought conditions. Our aim was to investigate the regulatory role of drought priming in establishing H_2_O_2_ homeostasis in leaves. We hypothesize that the enhanced drought resistance observed in soybean under subsequent severe stress is closely linked to the establishment of H_2_O_2_ homeostasis. This process is likely regulated by the dynamic activity of specific *WRKY* transcription factors and the modulation of carbohydrate metabolism in primed leaves.

## 2 Materials and methods

### 2.1 Plant material and growth conditions

A plastic greenhouse experiment was conducted in 2022 at the experimental station (32°02’ N, 118°50’ E) affiliated with Nanjing Agricultural University. The drought-tolerant soybean cultivar Kefeng 1 was sourced from the National Soybean Improvement Center at the same university. Soybean seeds were soaked in distilled water for 3–4h and then transferred to Petri dishes lined with moist filter paper for 3 days until germination. During this period, the filter paper was replaced daily to maintain moisture, and the germination process was conducted at 20 °C in the dark. Uniformly germinated seedlings were selected and planted in pots filled with organic growth substrate. Upon reaching the three-leaf stage, seedlings of uniform size were carefully removed from the pots, and their roots were rinsed thoroughly with deionized water. Subsequently, the plants were transferred to Hoagland nutrient solution and cultivated in a light incubator (Model GXZ-800D artificial climate chamber of the RDN series, supplied by Ningbo Southeast Instrument Co., LTD.). The temperature was held steady at 26 °C, with a light cycle consisting of 12h of illumination (30,000 l×) followed by 12h of darkness (0 l×). The Hoagland nutrient solution was used to acclimate hydroponic seedlings for five days, after which a continuous light treatment at 30,000 l× was applied for an additional two days to minimize the effects of biological rhythm on the experiment.

### 2.2 Experimental designs and treatments

**Figure 1:**
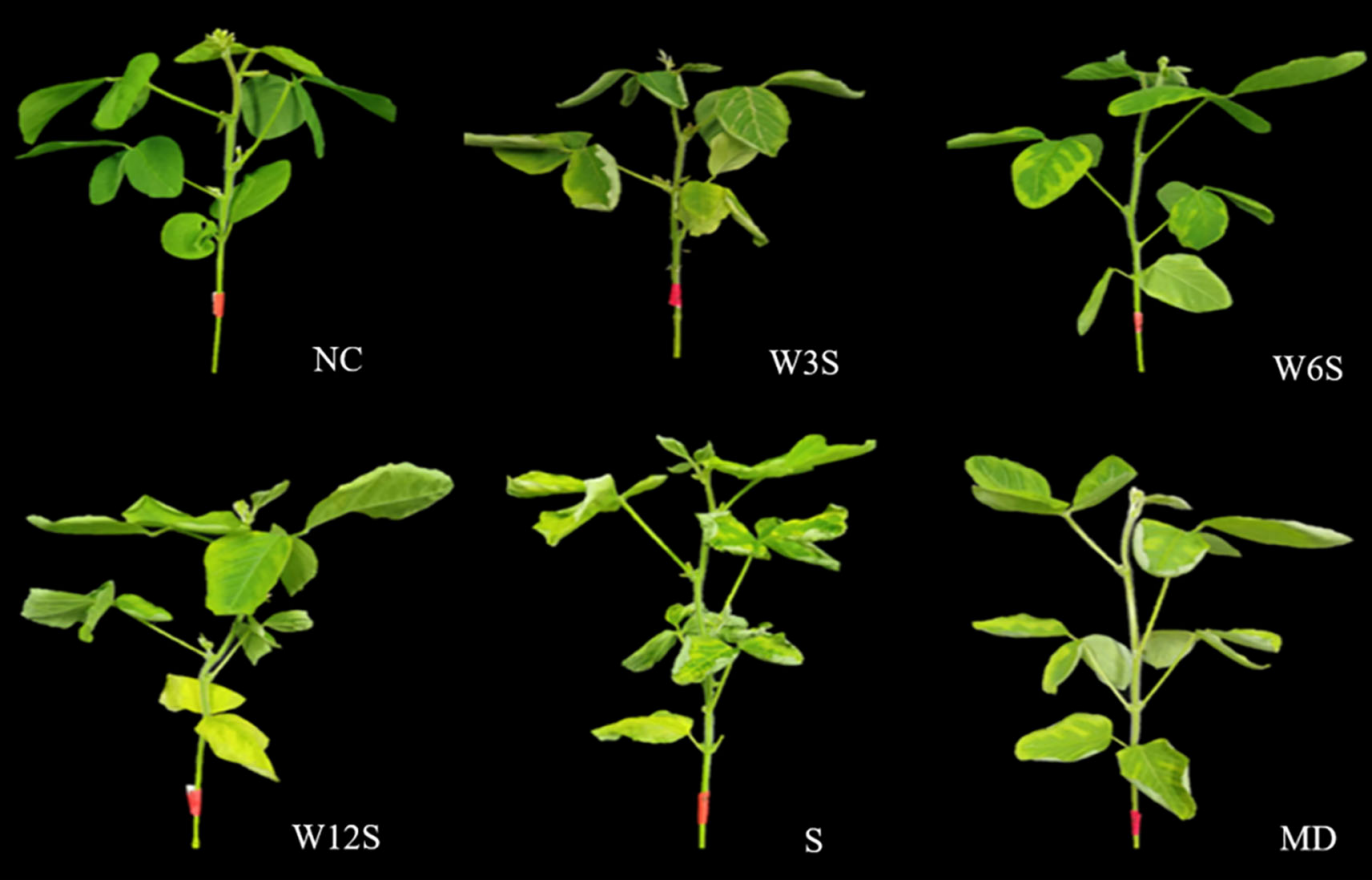
The performance of soybean seedlings at 24h under different drought treatments. NC represented normal cultivation. S indicated directly exposed to severe drought stress with 5% PEG6000 without any drought priming. MD represented maintained under mild drought stress with 0.5% PEG6000 throughout the experiment without subsequent severe drought stress. W3S indicated a 3h mild drought treatment with 0.5% PEG6000 prior to the severe drought treatment (5% PEG6000). W6S signified a 6h mild drought treatment with 0.5% PEG6000 before the severe drought treatment (5% PEG6000). W12S described as a scenario where the severe drought treatment (5% PEG6000) was preceded by a 12h mild drought treatment with 0.5% PEG6000.

Soybean seedlings were subjected to PEG6000 to simulate drought stress at the four-leaf stage. According to the results of Shen et al., 2024, six treatments were designed (**Table 1**) with normally cultivated plants (NC); directly exposed to severe drought stress with 5% PEG6000 without any drought priming (S); and maintained under mild drought stress with 0.5% PEG6000 throughout the experiment without subsequent severe drought stress (MD), serving as controls. Three priming scenarios were implemented: W3S, W6S, and W12S. In the W3S treatment, seedlings were exposed to 0.5% PEG6000 for 3h before undergoing severe drought treatment with 5% PEG6000. In the W6S treatment, seedlings experienced 6h of mild drought (0.5% PEG 6000) prior to the same severe drought treatment. For the W12S treatment, seedlings were pretreated with 0.5% PEG6000 for 12h before exposure to 5% PEG6000. The performance of soybean seedlings under the treatments was shown in **Figure 1**. To present the core results of this study more clearly and control the manuscript length, we excluded the analysis of two groups (Group S and Group MD) from the manuscript and only showed them in figures. Ternate compound leaves were sampled at 0, 3, 12, and 24h, with a minimum of 21 leaves collected from 21 plants per treatment at each time point. The water potentials of 0.5% PEG6000 and 5% PEG6000 were −0.09 MPa and −0.36 MPa, respectively. All samples were divided into three biological replicates and further partitioned into four portions. Two portions of the sample were quickly frozen in liquid nitrogen and maintained at −80 °C, which were prepared for subsequent enzyme activity assays and qRT-PCR detections. While the remaining segments were utilized to determine relative water content, relative conductivity, and metabolite levels.

**Table 1:** Treatment scheme.

Treatment	−12h	−6h	−3h	0h	3h	12h	24h
NC	0%	0%	0%	0%	0%	0%	0%
S	0%	0%	0%	5%	5%	5%	5%
MD	0.5%	0.5%	0.5%	0.5%	0.5%	0.5%	0.5%
W3S	0%	0%	0.5%	5%	5%	5%	5%
W6S	0%	0.5%	0.5%	5%	5%	5%	5%
W12S	0.5%	0.5%	0.5%	5%	5%	5%	5%

0%: Hoagland nutrient solution without PEG6000; 0.5%: Hoagland nutrient solution containing 0.5% PEG6000; 5%: Hoagland nutrient solution containing 5% PEG6000. NC represented normal cultivation. S indicated directly exposed to severe drought stress with 5% PEG6000 without any drought priming. MD represented maintained under mild drought stress with 0.5% PEG6000 throughout the experiment without subsequent severe drought stress. W3S indicated a 3h mild drought treatment with 0.5% PEG6000 prior to the severe drought treatment (5% PEG6000). W6S signified a 6h mild drought treatment with 0.5% PEG6000 before the severe drought treatment (5% PEG6000). W12S described as a scenario where the severe drought treatment (5% PEG6000) was preceded by a 12h mild drought treatment with 0.5% PEG6000.

### 2.3 Leaf relative water content and conductivity determination

We measured the relative water content of leaves according to the method outlined in Zhang (1990). The formula for calculating relative water content was as follows: Relative water content = (Wf−Wd)/(Wt−Wd)× 100%, where Wf represents the fresh weight of the sampled leaves, Wt denotes the weight of the leaves immersed in distilled water for 12h, and Wd indicates the constant weight of the dried leaves obtained through oven drying.

Fresh leaf samples weighing 0.3 g were rinsed with deionized water, and surface moisture was absorbed using filter paper. The leaves were then cut into thin strips measuring 1 cm × 2 mm, excluding the main vein, and placed into a 50-ml centrifuge tube. Each tube was filled with 20 ml of deionized water (ddH_2_O), covered, and soaked at room temperature for 12h. A conductivity meter was employed to measure the conductivity (R1) of the leaching solution. Subsequently, the tubes were heated in boiling water for 30 minutes, allowed to cool to room temperature, shaken, and the extract conductivity (R2) was measured again (Yu et al., 2023). The relative conductivity was calculated using the formula: Relative conductivity = (R1/R2) × 100%.

### 2.4 Metabolite’s determination

#### 2.4.1 MDA and H_2_O_2_ content determination

The content of malondialdehyde (MDA) in the leaves was measured utilizing a refined technique. To a 10 ml centrifuge tube, a mixture of 4 ml TCA-TBA and 2 ml of enzyme solution was introduced. After a 20-min treatment in a boiling water bath, the mixed samples were centrifuged with a 10-min run at 4,000 rpm (Rotor F1010, Beckman Coulter). The supernatant was then collected for analysis. Following this, the MDA levels (nmol g^−1^ FW) were quantified at wavelengths of 450 nm, 532 nm, and 600 nm. For the extraction and measurement of H_2_O_2_ in soybean leaves, the H_2_O_2_ (μmol g^−1^ FW) assay kit originated from Nanjing Jiancheng Bioengineering Institute, located in Nanjing, China.

#### 2.4.2 Sucrose, glucose, fructose, and starch content determination

The contents of total soluble sugar, sucrose, and fructose were assessed using the methodology proposed by Gao et al. (2012). The resorcinol color development method was employed, whereby 100 μl of the extract solution was introduced into a centrifuge tube. Subsequently, 50 μl of 2 M NaOH was added, and the mixture was boiled for 5 min. After cooling under running water, 700 μl of 30% HCl and 200 μl of a 0.1% resorcinol solution were added sequentially. The resulting mixture was then maintained in a water bath at 80 °C for 10 min. The absorbance at a wavelength of 480 nm was measured to determine the sucrose content.

To determine the fructose content, 100 μl of the extract was mixed with 100 μl of a 0.1% resorcinol solution. Subsequently, 700 μl of 30% HCl was added to the mixture, which was then mixed well. Following a 10-min incubation at 80 °C, absorbance was measured at 480 nm. For glucose content determination, 200 μl of the extract solution was mixed with 400 μl of the reaction solution, which contained 10 U ml^−1^ of glucose oxidase, 0.1 mg ml^−1^ of o-anisidine-HCl, and horseradish peroxidase. Incubation of the mixture was carried out at 30 °C for 5 min. To terminate the reaction, 5 M sulfuric acid (H_2_SO_4_) was immediately added, and the absorbance was measured at a wavelength of 505 nm. The fructose and glucose contents were calculated according to the standard curve. The sum of the fructose and glucose contents represents the total hexose content. The starch content was determined using the anthrone-sulfuric acid method. Specifically, 100 μl of the extract was mixed with 3 μl of anthrone reagent (150 μl of anthrone dissolved in 100 μl of 10 mol L^−1^ H_2_SO_4_) and maintained at 90 °C for 15 min. The absorbance value at 620 nm was determined, with starch content subsequently calculated based on the established standard curve.

### 2.5 Enzymatic analyses

#### 2.5.1 SOD, POD, and CAT activity assessment

Fresh leaf samples weighing 0.3 g were placed in a pre-cooled mortar. Add quartz sand and grind them into a homogenate with 5 mL of 50 mmol L^−1^ extraction solution [Tris-HCl (pH 7.0), 20% glycerin, 1 mmol L^−1^ DTT, 1 mmol L^−1^ EDTA, 1 mmol L^−1^ ASA, 1 mmol L^−1^ GSH, and 5 mmol L^−1^ MgCl_2_] on an ice bath. Rinse the homogenate, transfer it to a centrifuge tube, and perform centrifugation at 16,000 rpm (Rotor F1010, Beckman Coulter) for 20 min under 4 °C. The supernatant thus obtained was regarded as the crude enzyme extract.

The SOD activity (μmol mg^−1^ FW min^−1^) was assessed using the nitrogen blue tetrazole (NBT) method by monitoring the inhibition of photochemical reduction of NBT as described in Lei et al. (2006). The guaiacol method was utilized to assess POD activity (μmol mg^−1^ FW min^−1^). It was estimated as the decomposition rate of H_2_O_2_ by POD, with guaiacol as hydrogen donor, by measuring the absorbance change rate at 470 nm. CAT activity (μmol mg^−1^ FW min^−1^) was evaluated using the ultraviolet absorption method (Hu et al., 2016).

#### 2.5.2 SuSy, SPS, and INV activity assessment

For the extraction and activity determination of sucrose synthase- and decomposition-related enzymes, 0.5 g of leaves was combined with grinding beads in a 10-ml centrifuge tube. The mixture was ground into a powder using a grinder after being rapidly frozen with liquid nitrogen. Subsequently, 5 ml of extraction solution [50 mmol L^−1^ Hepes-NaOH (pH 7.5), 5 mmol L^−1^ MgCl_2_, 1 mmol L^−1^ EDTA-Na_2_, 2.5 mmol L^−1^ DTT, and 0.5% BSA] was added, and the mixture was thoroughly shaken to ensure even distribution. Finally, the composite samples were centrifuged at 12,000 rpm (Rotor F1010, Beckman Coulter) for 20 min to yield the supernatant as the crude enzyme extract.

The SPS activity was determined using the method of Pavlinova et al. (2002), with some modifications. 350 μl reaction mixtures (50 mM extraction buffer, 10 mM MgCl_2_, 50 mM UDP-glucose, and 50 mM fructose-6-P) and 200 μl extract were incubated at 30 °C for 30 min. Add 100 μl of 2 M NaOH to the reaction system, then heat the mixture at 100 °C for 10 min to terminate the reaction. Subsequently, mix the system with 1 ml of 0.1% (w/v) resorcin dissolved in 95% ethanol and 3.5 ml of 30% (w/v) HCl, followed by incubation at 80 °C for 10 min. Finally, determine the absorbance of the resultant supernatant at 480 nm.

The method and procedure for determining the SuSy activity in the synthesis direction were identical to those for SPS, with the only modification being the substitution of F-6-P with fructose. The SuSy activity (decomposition direction) was referred to in Zinselmeier et al. (1995), with some modifications. The 650 μl of reaction mixture contained 2 mM of UDP, 100 mM of sucrose, 20 mM of Pipes-NaOH (pH 6.5), 50 mM of MgCl_2_, and 200 μl of extract. The reaction was incubated for 30 min at 30 °C). After adding 250 μl of 0.5 M Tricine-KOH (pH 8.3), the reaction was terminated by heating at 100 °C for 10 min. Fructose content was quantified based on absorbance measurements at 540 nm and a pre-established standard curve.

The A-INV activity was referenced in Pavlinova et al. (2002) with some modifications. A total of 0.1 ml of crude enzyme solution and 2.4 ml of reaction solution (comprising 2.2 ml of 0.2 mol L^−1^ acetoacetate-potassium acetate buffer at pH 5.0 and 0.2 ml of 1 mol L^−1^ sucrose) were added to a 10 ml centrifuge tube. The mixture was incubated in a water bath at 30 °C for 30 min, after which 1 ml of DNS reagent was added to terminate the reaction. Following this, the mixture was subjected to a boiling water bath for 5 min, cooled on ice, and then analyzed using an enzyme marker at 540 nm to compare color and record the absorbance value, ultimately allowing for the calculation of enzyme activity. The method and procedure for determining the activity of N-INV were identical to those for A-INV, except that the pH of the 0.2 mol L^−1^ potassium acetyl-acetate buffer was adjusted to 7.5.

### 2.6 Real-time quantitative PCR analysis

Total RNA was extracted from soybean leaves using the Steady Pure Plant RNA Extraction Kit provided by Changsha Eckerui Biotechnology Co., Ltd. The concentration and integrity of the purified RNA were measured using agarose gel electrophoresis as well as a NanoDrop One spectrophotometer. The RNA was subsequently stored at −80 °C. For RNA reverse transcription, the Evo M-MLV Reverse Transcription Kit from Changsha Ecorui Biotechnology Co., Ltd. was utilized. The reaction conditions included an initial incubation at 37 °C for 15 minutes to facilitate reverse transcriptase activity, followed by a 5-min inactivation of reverse transcriptase at 85 °C, with a final incubation maintained at 4 °C. The resulting retrotranscripts were stored at −20 °C. qRT-PCR was conducted using the CFX96 real-time fluorescent quantitative PCR instrument with the SYBR Green Pro Taq HS premixed qPCR kit from Changsha Ecoray Biotechnology Co., Ltd. Primers for GmUBI3 and GmEF1, used as reference genes, were designed using Primer software and synthesized by Jinsilui Biotechnology Co., Ltd. (Nanjing). The primer sequences are presented in **Table 2**. Based on recent transcriptomic and functional studies (Zhang et al., 2025; Zhu et al., 2023) and our previous study (Shen, 2024), we selected *WRKY* transcription factors (Gm*WRKY33a/b* and Gm*WRKY12*) along with key antioxidant enzymes (catalase, peroxidase) and hexose metabolism-related genes, as these components were reported to coordinate reactive oxygen species (ROS) signaling and stress memory.

**Table 2:** qRT-PCR primer sequence list.

Measuring index	Gene	Reference	Primer sequence (5’→3’)
WRKY	GmWRKY33(a)	Yang et al., 2025	F: CAATTCATCAAACTTCTTCGTCCTG R: GGCCTCATTTCCAAGGTCA
	GmWRKY12	Shi et al., 2018	F: CTACAAATGCACACAACCCACT R: TCGCAATGTGTATGAGTACCCT
	GmWRKY27	Wang et al., 2015	F: GAACCCAATGAATCCGTGTCTTGC R: TTCCTCGTTTTCCAGCGTGTCA
	GmWRKY33(b)	Pruthi et al., 2024	F: AACCCTGGAAGTTTTGGCTA R: AGCATCTGTGTATTGCGCTT
	GmUBI3	De La Torre and Finer, 2015	F: GTGTAATGTTGGATGTGTTCCC R: ACACAATTGAGTTCAACACAAACCG
Antioxidant-related enzyme gene	GmSOD	Hussain et al., 2023	F: GTGAAGGCTGTGGCAGTTCT R: GGTAGTGTCCCCCAAGGCAT
	GmPOD		F: TCAGCAACACTGGAAACCCT R: GCATTCTGGGGGCATCTTG
	GmCAT	Khan et al., 2026	F: CACCAAGACCACAACGCAAG R: GGGCAACGCAAAACGAAAGA
	GmEF1	Gao et al., 2019	F: GCTGCCAACTTCACATCCCA R: TCAAGGACTGGTGCGTATCC
	GmA-INV	Freymuth and Fitzsimons, 2017	F: TCAGGCAAATGATGTCCGCA R: CAACCGTCCTTCCACCTTGA
Sucrose metabolism-related enzyme genes	GmN-INV	Zhang et al., 2024	F: TGCTTGTCAGAGGGGTTTGAT R: TTCGGCGTCATCCTGTTTCA
	GmSPS	Shen et al., 2023	F:TGAATACCGTTGGGGTGGAG R: TCCTGGCTTTCGCACTTTGA
	GmSuSy	Cui et al., 2023	F: ATTTTCGTTGGATTGCTGCCC R: GCACCTTGTGTGTCTGCTAT
	GmEF1	Gao et al., 2019	F: GCTGCCAACTTCACATCCCA R: TCAAGGACTGGTGCGTATCC

### 2.7 Statistical analysis

Data were analyzed using a one-way analysis of variance (ANOVA), and the differences between mean values were assessed using Student’s t-test. All statistical analyses were performed using SPSS 26.0 software (IBM Corporation, USA). Significant differences between the compared groups were indicated by distinct lowercase letters. Figures were generated using Origin 2023 (OriginLab, USA).

## 3 Results

### 3.1 Relative water content and relative conductivity in leaves

**Figure 2:**
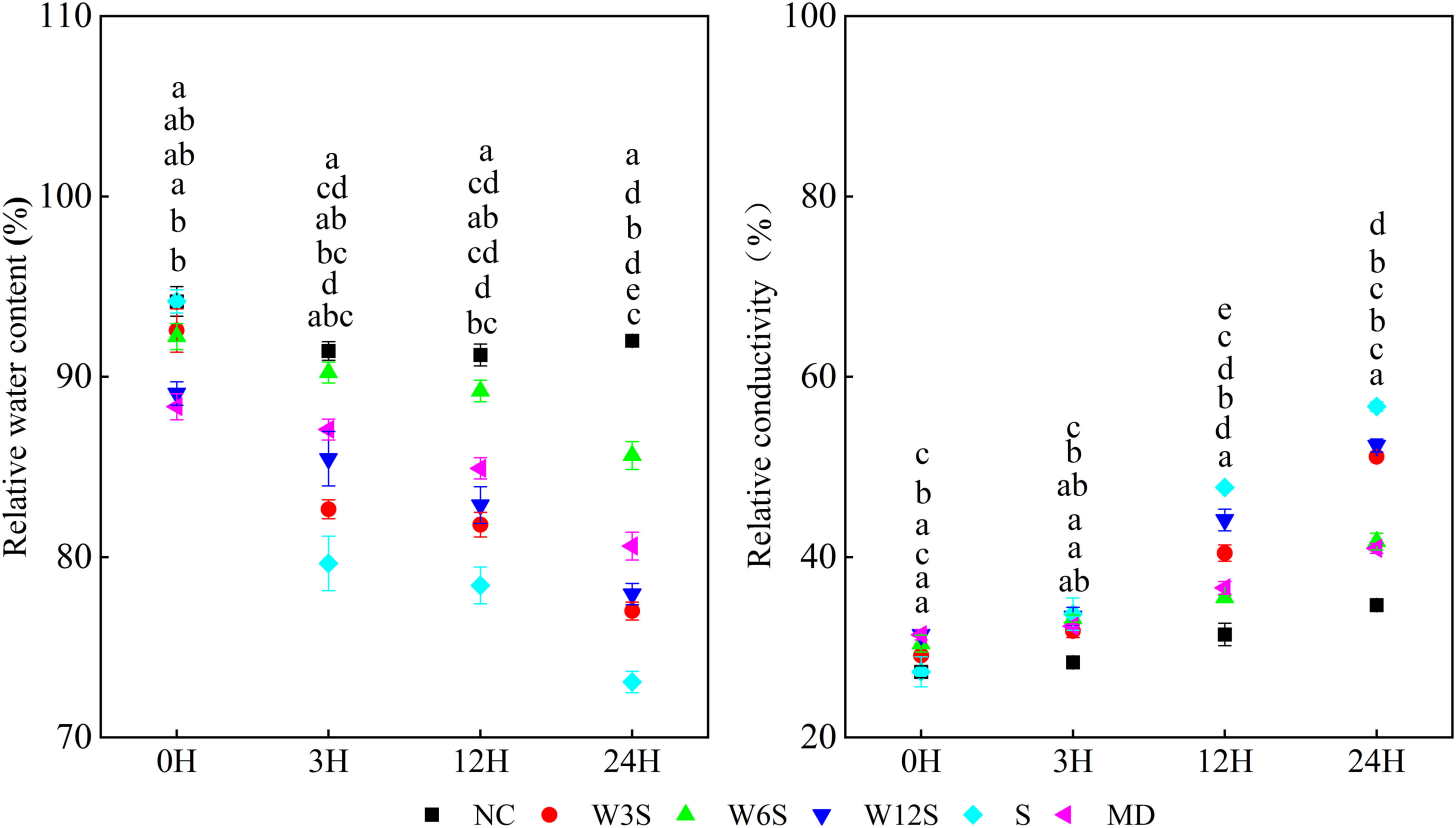
Changes of relative water content and relative conductivity in soybean leaves under different treatments. NC represented normal cultivation. S indicated directly exposed to severe drought stress with 5% PEG6000 without any drought priming. MD represented maintained under mild drought stress with 0.5% PEG6000 throughout the experiment without subsequent severe drought stress. W3S indicated a 3h mild drought treatment with 0.5% PEG6000 prior to the severe drought treatment (5% PEG6000). W6S signified a 6h mild drought treatment with 0.5% PEG6000 before the severe drought treatment (5% PEG6000). W12S described as a scenario where the severe drought treatment (5% PEG6000) was preceded by a 12h mild drought treatment with 0.5% PEG6000. The data were derived from three biological replicates and are presented in the form of standard values ± standard errors. Different letters were significantly different at the 0.05 probability level.

The relative water content of all treated leaves showed a decreasing trend (**Figure 2**). The relative water content of leaves treated with S and MD was lower than NC treatment. Compared to the NC group, the relative water content of leaves under W6S treatment did not show significant differences at 0, 3, and 12h but was significantly reduced at 24h. For W3S and W12S treatments, the relative water content of leaves significantly decreased during the time period from 3 to 24h, with a decline of 9.6%–16.3% and 6.5%–15.3%, respectively.

In terms of the relative conductivity of the leaves, all treatment groups exhibited an upward trend. The relative conductivity of the leaves under the W3S treatment significantly increased by 6.6%, 12.3%, 28.8%, and 47.5% at 0, 3, 12, and 24h, respectively, when compared to the NC group. Meanwhile, the W6S treatment resulted in significant increases of 11.5%, 17.1%, 13.0%, and 20.4% at the same time points. Additionally, under the W12S treatment, the increases in relative conductivity of the leaves were 15.1%, 18.0%, 40.4%, and 51.1%, respectively. For S, the increases were 0%, 18.9%, 52.0%, and 63.6%, while under MD, the increases were 15.1%, 14.2%, 16.5%, and 18.2%.

### 3.2 Changes in H_2_O_2_ and MDA content in leaves

**Figure 3:**
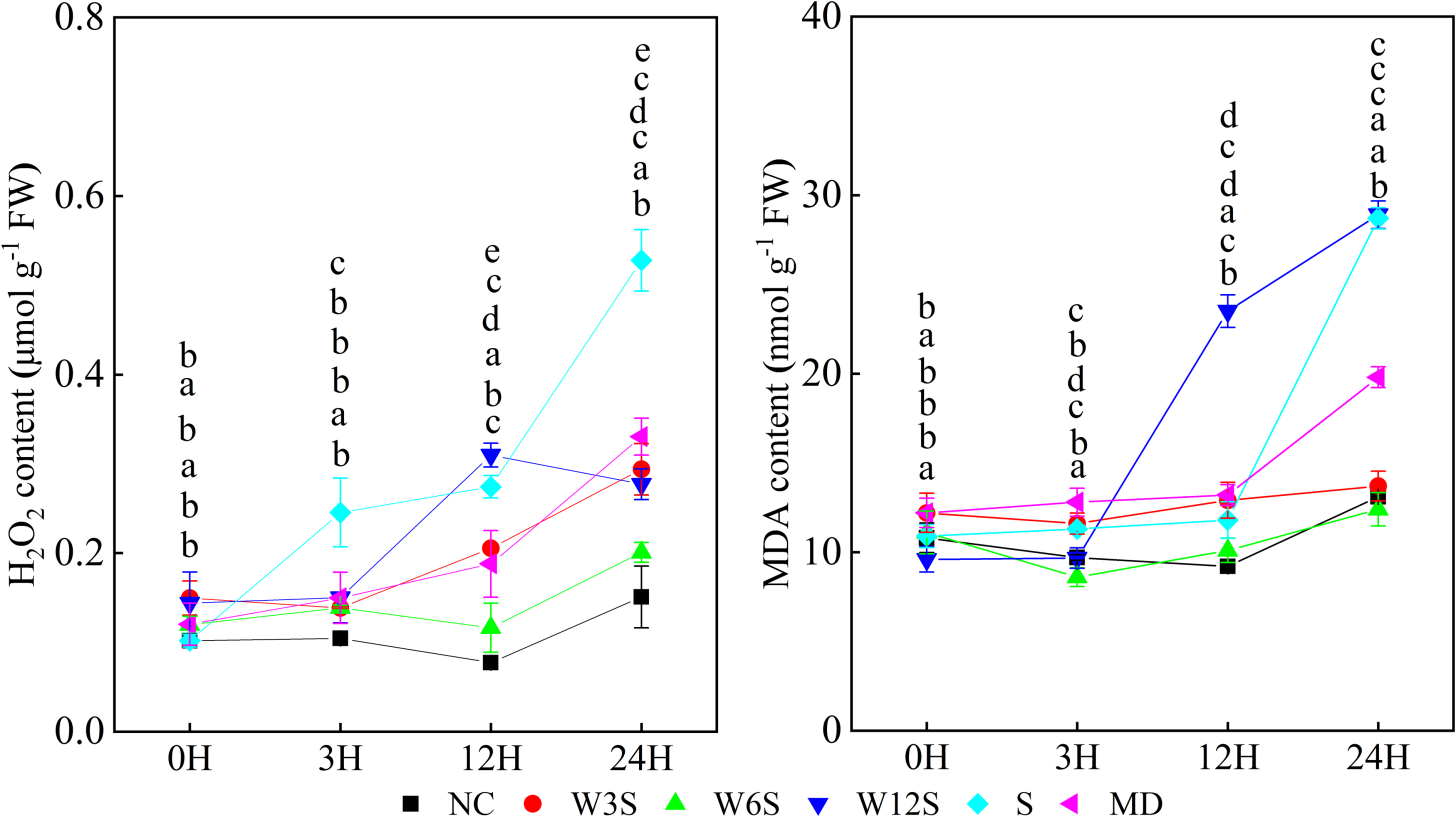
Changes of H_2_O_2_ content and MDA content in soybean leaves under different treatments. H means hours. NC represented normal cultivation. S indicated directly exposed to severe drought stress with 5% PEG6000 without any drought priming. MD represented maintained under mild drought stress with 0.5% PEG6000 throughout the experiment without subsequent severe drought stress. W3S indicated a 3h mild drought treatment with 0.5% PEG6000 prior to the severe drought treatment (5% PEG6000). W6S signified a 6h mild drought treatment with 0.5% PEG6000 before the severe drought treatment (5% PEG6000). W12S described as a scenario where the severe drought treatment (5% PEG6000) was preceded by a 12h mild drought treatment with 0.5% PEG6000. The data were derived from three biological replicates and are presented in the form of standard values ± standard errors. Different letters were significantly different at the 0.05 probability level.

Following drought stress, there was a notable increase in the concentration of H_2_O_2_ within the leaves (**Figure 3**). Specifically, the H_2_O_2_ levels under W6S treatment showed substantial rises of 16.6%, 17.5%, 18.4%, and 19.6% at 0, 3, 12, and 24h, respectively, when compared to the NC treatment. However, despite the significant increase, the overall trend remained aligned with that seen in the NC group. This suggests that during this time frame, the H_2_O_2_ levels in the leaves were maintained in a dynamic equilibrium state. In contrast, while the H_2_O_2_ content in the leaves under the W3S and W12S treatments also increased significantly from 3h to 24h, a dynamic balance akin to what was noted in the W6S treatment was not evident when comparing the NC group. When assessed against the NC treatment, the MDA levels in the leaves of the W3S treatment group showed a significant increase at 3h and 12h but returned to levels comparable to NC at 24h. The W6S treatment group did not exhibit significant variations in comparison to the NC group from 3h to 24h. Conversely, the MDA content within the W12S treatment group markedly increased at 12 hours and 24h when contrasted with the NC group, demonstrating increases of 155.4% and 120.6%, respectively. In addition, the increases were 7.9%, 11.7%, 32.3%, and 60.7% under MD. Since the S treatment commenced at 0H, the indicators were not influenced at this sampling time when compared to NC. However, the H_2_O_2_ content under S at 3H, 12H, and 24H significantly increased, reaching the highest levels among all treatments.

Although disparities in H_2_O_2_ and MDA levels existed between the W6S treatment group and the NC group, the fluctuations in H_2_O_2_ and MDA concentrations under W6S treatment were consistent with those under NC, suggesting that the establishment of a steady-state equilibrium was formed under W6S. Thus, we further investigated and compared the regulatory mechanisms that contribute to the formation of this steady state in the W6S treatment.

### 3.3 Alterations in antioxidant oxidase activity in leaves

**Figure 4:**
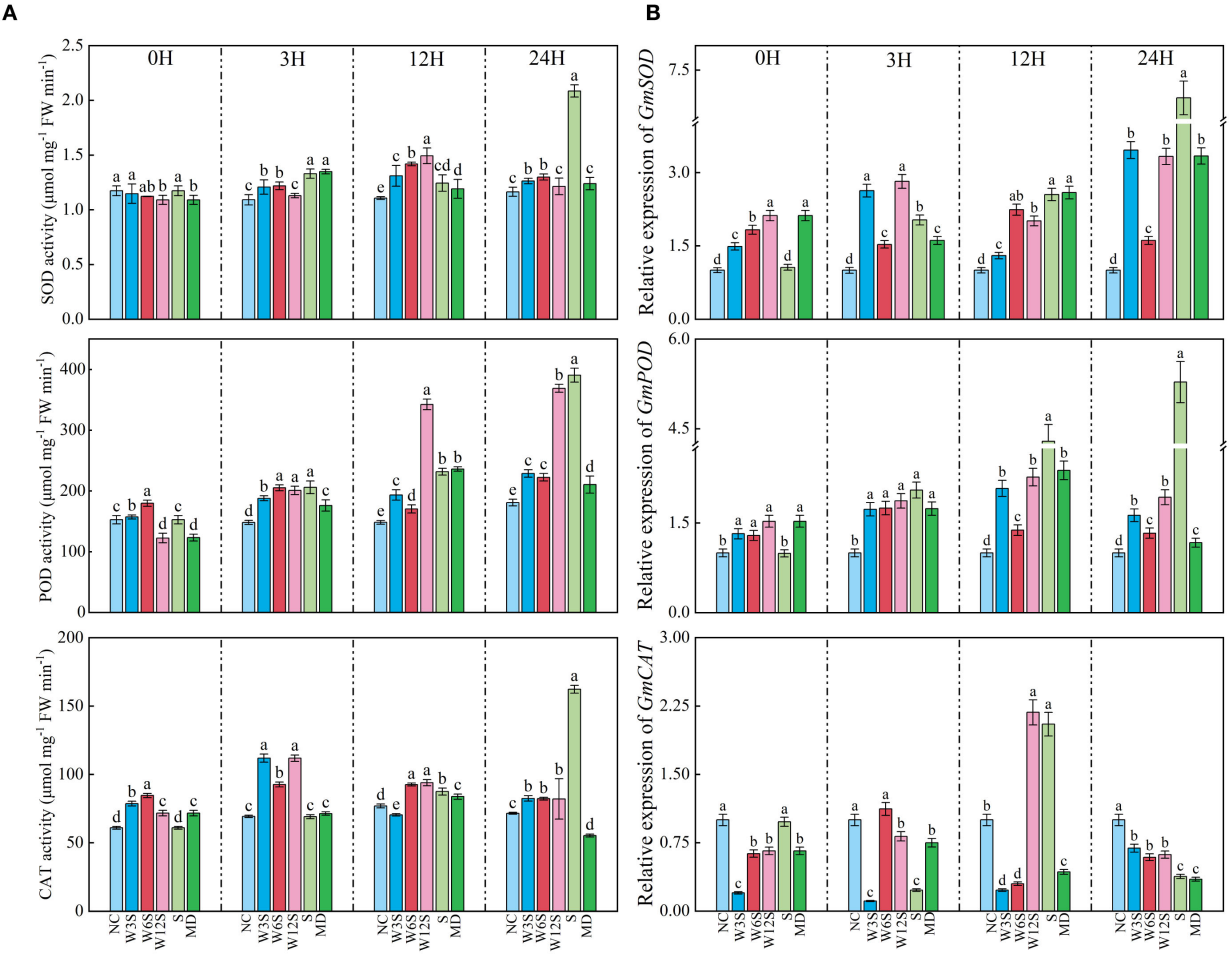
**(a)** Changes of SOD, POD, and CAT activity content in leaves under different treatments; **(b)** Relative expressions of *GmSOD*, *GmPOD*, and *GmCAT* in leaves under different treatments. H means hours. NC represented normal cultivation. S indicated directly exposed to severe drought stress with 5% PEG6000 without any drought priming. MD represented maintained under mild drought stress with 0.5% PEG6000 throughout the experiment without subsequent severe drought stress. W3S indicated a 3h mild drought treatment with 0.5% PEG6000 prior to the severe drought treatment (5% PEG6000). W6S signified a 6h mild drought treatment with 0.5% PEG6000 before the severe drought treatment (5% PEG6000). W12S described as a scenario where the severe drought treatment (5% PEG6000) was preceded by a 12h mild drought treatment with 0.5% PEG6000. The data were derived from three biological replicates and are presented in the form of standard values ± standard errors. Different letters were significantly different at the 0.05 probability level.

The W6S treatment significantly enhanced the activities of SOD, POD, and CAT in the leaves (**Figure 4a**). Compared to the NC treatment, the SOD activity under W6S treatment increased significantly by 11.7%, 28.1%, and 11.6% at 3h, 12h, and 24h, respectively. The activities of POD and CAT exhibited significant increases following the onset of drought, with POD activity increasing significantly by 17.8%, 38.4%, 14.9%, and 23.0% at 0h, 3h, 12h, and 24h, respectively. Meanwhile, CAT activity showed significant increases of 38.8%, 33.7%, 20.3%, and 15.0%, respectively.

In comparison to the NC treatment, W6S treatment also significantly enhanced the relative expression levels of *GmSOD* and *GmPOD* in the leaves (**Figure 4b**). At 0h, 3h, 12h, and 24h, the relative expression of *GmSOD* increased by 0.83-fold, 0.53-fold, 1.24-fold, and 0.61-fold, respectively. In contrast, *GmPOD* expression increased by 0.29-fold, 0.75-fold, 0.38-fold, and 0.33-fold, respectively. However, regarding the relative expression of *GmCAT*, W6S treatment did not appear to effectively enhance its relative expression, as the relative expression of *GmCAT* was significantly downregulated at 0h, 12h, and 24h. Based on the results, we believed that under W6S treatment, *GmSOD* and *GmPOD* primarily play a role in regulating the changes in the antioxidant capacity of the leaves.

### 3.4 Relative expression of WRKY transcription factors in leaves

**Figure 5:**
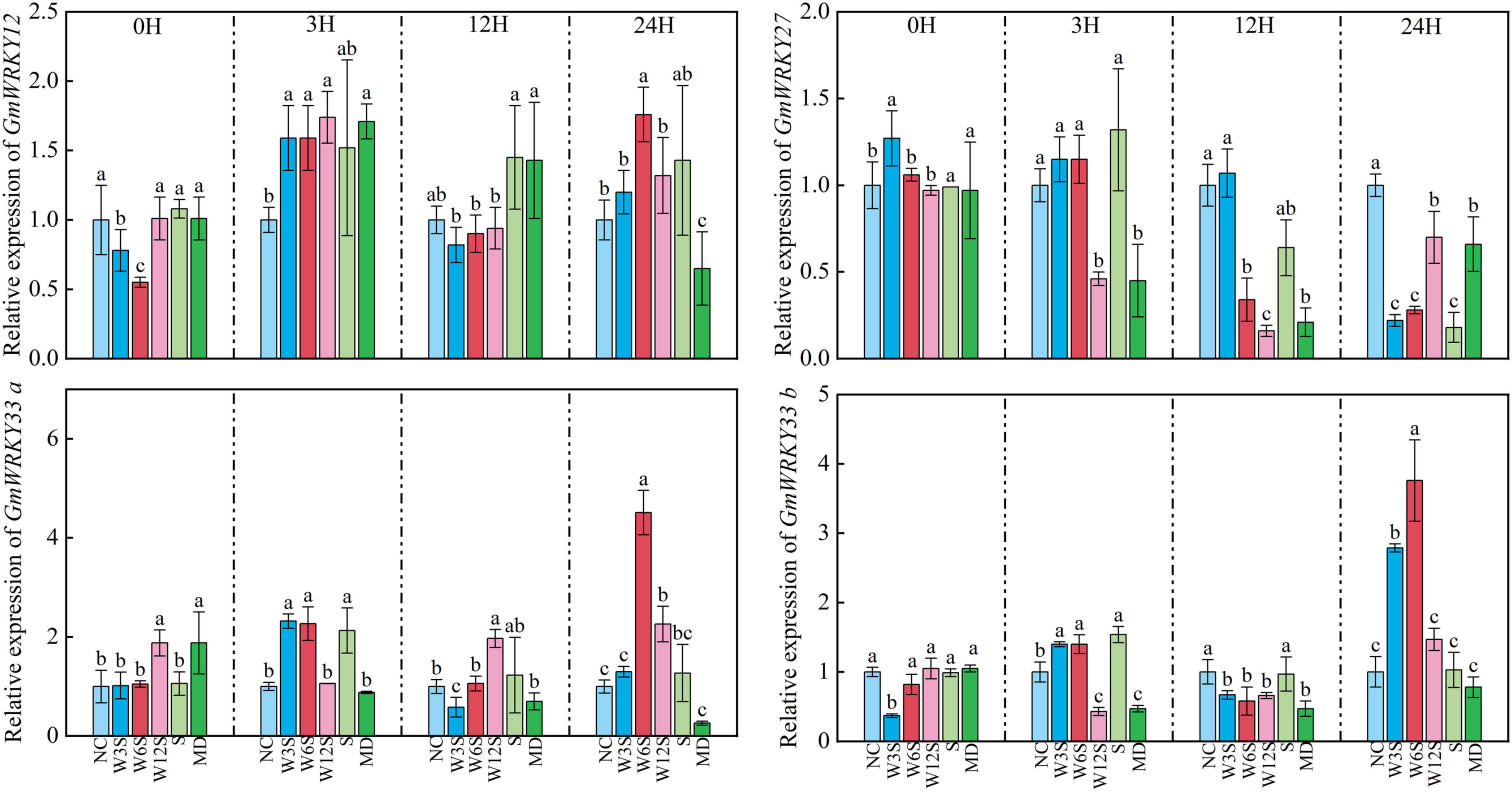
Relative expression of *GmWRKY33a*, *GmWRKY33b*, *GmWRKY12*, and *GmWRKY27* in leaves under different treatments. H means hours. NC represented normal cultivation. S indicated directly exposed to severe drought stress with 5% PEG6000 without any drought priming. MD represented maintained under mild drought stress with 0.5% PEG6000 throughout the experiment without subsequent severe drought stress. W3S indicated a 3h mild drought treatment with 0.5% PEG6000 prior to the severe drought treatment (5% PEG6000). W6S signified a 6h mild drought treatment with 0.5% PEG6000 before the severe drought treatment (5% PEG6000). W12S described as a scenario where the severe drought treatment (5% PEG6000) was preceded by a 12h mild drought treatment with 0.5% PEG6000. The data were derived from three biological replicates and are presented in the form of standard values ± standard errors. Different letters were significantly different at the 0.05 probability level.

To clarify the regulatory roles of *GmWRKYs* in the formation of leaf homeostasis under varying treatment conditions, we selected *GmWRKY12*, *GmWRKY27*, *GmWRKY33a*, and *GmWRKY33b* for expression analysis (**Figure 5**). The results indicated that under W6S treatment, the relative expression levels of *GmWRKY33a*, *GmWRKY33b*, and *GmWRKY12* were significantly upregulated at both 3h and 24h; specifically, *GmWRKY33a* exhibited increases of 1.27-fold and 3.51-fold at 3h and 24h, respectively, while *GmWRKY33b* showed increases of 0.40-fold and 2.76-fold at the same time points. The relative expression of *GmWRKY12* increased by 0.59-fold and 0.76-fold at 3h and 24h, respectively. However, at 12h, their relative expression levels decreased to the baseline observed in the NC treatment, or even lower. This finding aligns with results indicating that leaves can establish a new H_2_O_2_ homeostasis between 3h and 24h. Additionally, we noted that the changes in *GmWRKYs* expression under W3S treatment were comparable to those observed under W6S treatment; however, the upregulation of WRKY TFs at 24h under W3S treatment was significantly lower than that observed under W6S treatment. Regarding the relative expression of *GmWRKY27*, it appears that under W6S treatment, there is no significant difference in its relative expression at 3h compared to NC, while at 12h and 24h, it is significantly downregulated. This suggested that after experiencing drought treatment, *GmWRKY27* appeared to play a negative regulatory role in the formation of new H_2_O_2_ homeostasis in the leaves.

### 3.5 Alterations in sucrose metabolite-related substances in leaves

**Figure 6:**
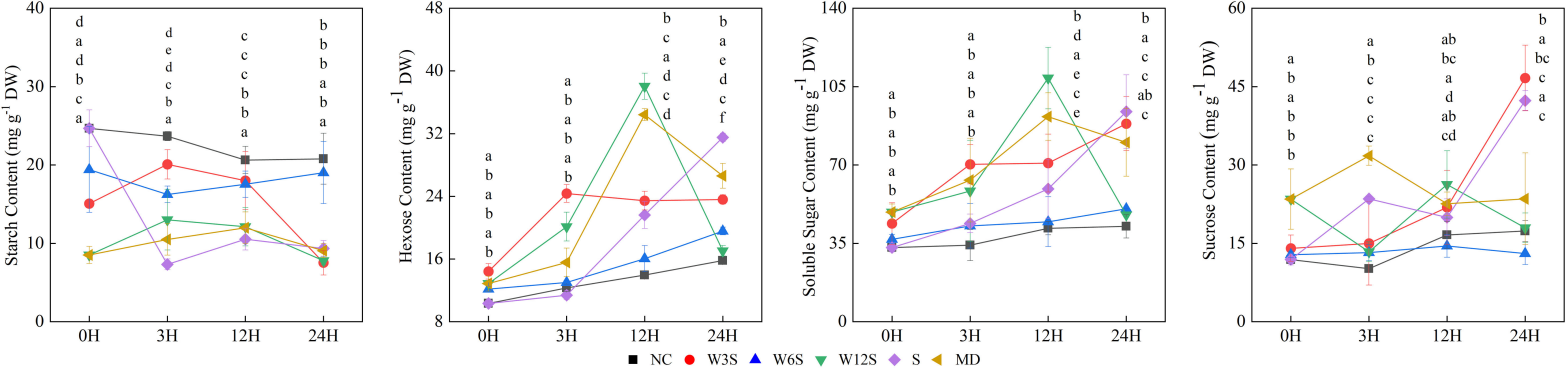
Changes of soluble sugar content and starch content in leaves under different treatments. H means hours. NC represented normal cultivation. S indicated directly exposed to severe drought stress with 5% PEG6000 without any drought priming. MD represented maintained under mild drought stress with 0.5% PEG6000 throughout the experiment without subsequent severe drought stress. W3S indicated a 3h mild drought treatment with 0.5% PEG6000 prior to the severe drought treatment (5% PEG6000). W6S signified a 6h mild drought treatment with 0.5% PEG6000 before the severe drought treatment (5% PEG6000). W12S described as a scenario where the severe drought treatment (5% PEG6000) was preceded by a 12h mild drought treatment with 0.5% PEG6000. The data were derived from three biological replicates and are presented in the form of standard values ± standard errors. Different letters were significantly different at the 0.05 probability level.

Compared to the NC group, the soluble sugar levels in the W6S group showed a slight increase from 0h to 24h; however, this change did not reach statistical significance (**Figure 6**). In terms of sucrose content, the W6S group exhibited a significant decrease compared to the NC group at 12 and 24h, with reductions of 12.7% and 24.9%, respectively. The hexose sugar content in the leaves significantly increased by 17.6%, 15.0%, and 23.9% at 0h, 12h, and 24h, respectively, while no significant difference was observed at 3h compared to the NC group. This experiment also found that the starch content in the leaves of all drought treatment groups was significantly lower compared to the NC group. In the W6S group, the reductions in starch content from 0h to 24h were 21.4%, 31.4%, 15.0%, and 56.6%, respectively.

### 3.6 Changes in enzyme activities related to sugar metabolism in soybean leaves

**Figure 7:**
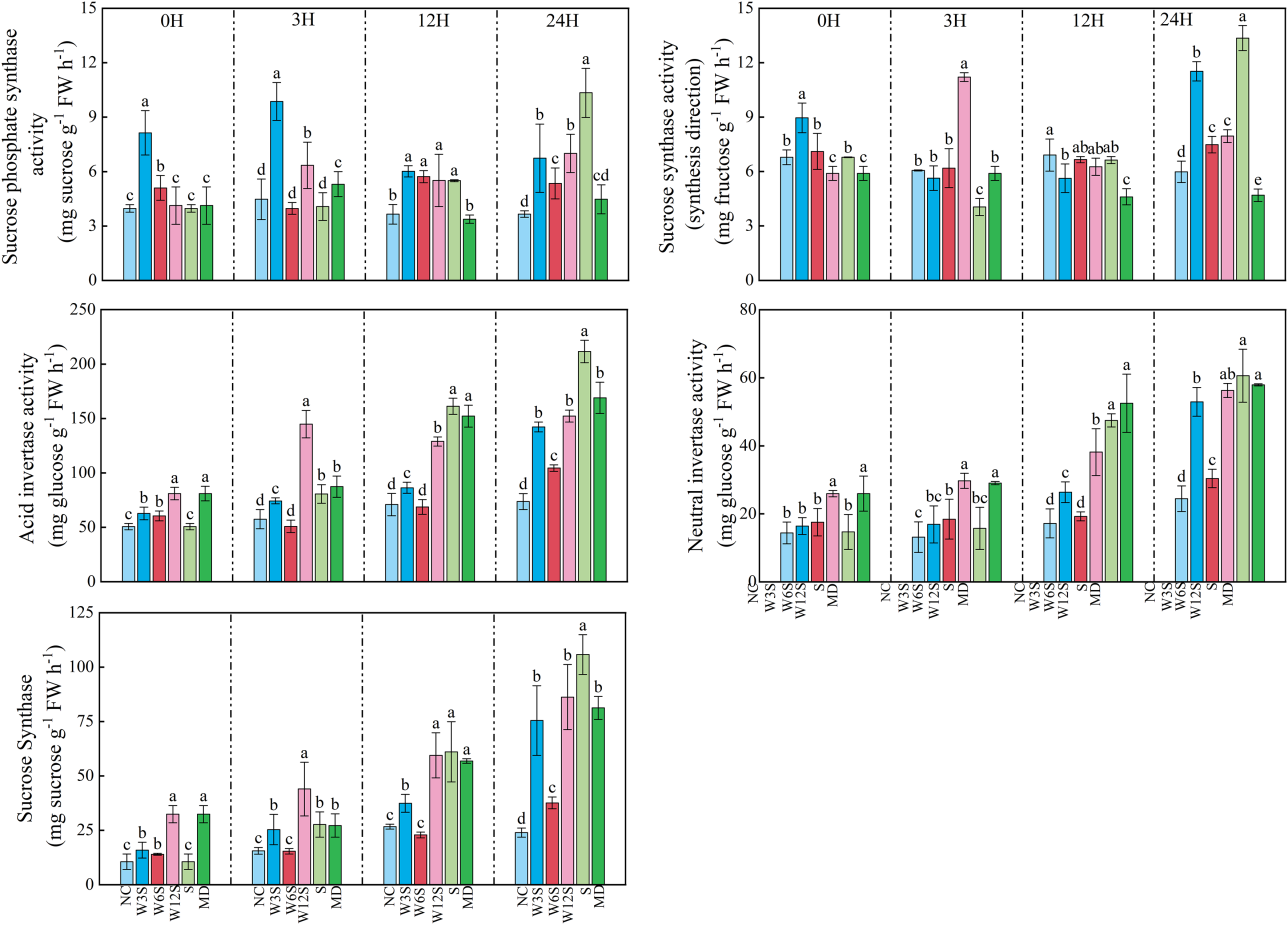
Changes of carbohydrate-related enzymes in leaves under different treatments. H means hours. NC represented normal cultivation. S indicated directly exposed to severe drought stress with 5% PEG6000 without any drought priming. MD represented maintained under mild drought stress with 0.5% PEG6000 throughout the experiment without subsequent severe drought stress. W3S indicated a 3h mild drought treatment with 0.5% PEG6000 prior to the severe drought treatment (5% PEG6000). W6S signified a 6h mild drought treatment with 0.5% PEG6000 before the severe drought treatment (5% PEG6000). W12S described as a scenario where the severe drought treatment (5% PEG6000) was preceded by a 12h mild drought treatment with 0.5% PEG6000. The data were derived from three biological replicates and are presented in the form of standard values ± standard errors. Different letters were significantly different at the 0.05 probability level.

The W6S treatment typically promoted SPS activity in the leaves; however, the level of this enhancement was notably lower compared to that observed with the W3S treatment (**Figure 7**). When assessing the NC group, the SPS activity in leaves subjected to W6S treatment demonstrated significant increases of 28.5%, 46.1%, and 56.8% at 0h, 12h, and 24h, respectively. In terms of the changes in SuSy activity toward synthesis, this research indicated that under W6S treatment, no significant differences in SuSy activity were noted at 0h, 3h, and 12h relative to the NC group, with a marked increase of 25.1% occurring exclusively at 24h.

A-INV, N-INV, and SuSy successfully hydrolyze sucrose into fructose and glucose for subsequent metabolic processes. This investigation showed that as drought stress continued, the activities of these three enzyme types generally trended upwards, while the variation in A-INV remained relatively stable under W6S conditions. In comparison to the NC group, the activity of A-INV in the leaves significantly increased by 41.7% at 24h under W6S, while N-INV activity exhibited significant increments at both 3h and 24h, specifically increases of 39.9% and 24.3%, respectively. Regarding the changes in the cleavage direction of SuSy, this study confirmed that the SuSy activity in the leaves under W6S treatment increased by only 43.4% at 24h.

### 3.7 Changes in the relative expression levels of sucrose metabolism-related genes

**Figure 8:**
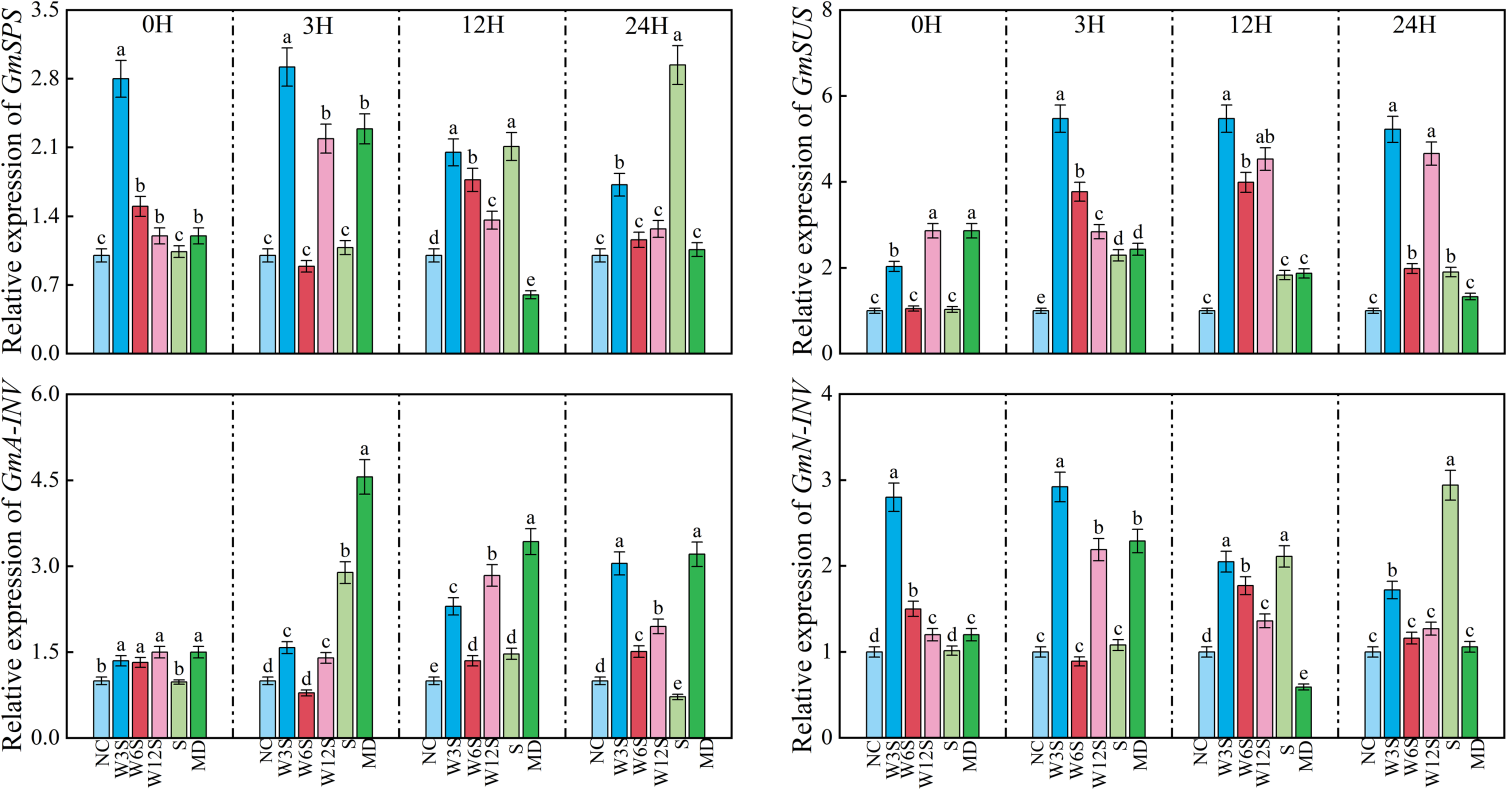
Changes of the relative expression levels of sucrose metabolism-related genes in leaves under different treatments. H means hours. NC represented normal cultivation. S indicated directly exposed to severe drought stress with 5% PEG6000 without any drought priming. MD represented maintained under mild drought stress with 0.5% PEG6000 throughout the experiment without subsequent severe drought stress. W3S indicated a 3h mild drought treatment with 0.5% PEG6000 prior to the severe drought treatment (5% PEG6000). W6S signified a 6h mild drought treatment with 0.5% PEG6000 before the severe drought treatment (5% PEG6000). W12S described as a scenario where the severe drought treatment (5% PEG6000) was preceded by a 12h mild drought treatment with 0.5% PEG6000. The data were derived from three biological replicates and are presented in the form of standard values ± standard errors. Different letters were significantly different at the 0.05 probability level.

In the W6S group, the relative expression of *GmSPS*, *GmA-INV*, and *GmN-INV* in the leaves showed significant changes (**Figure 8**). Notably, these three types of genes were significantly upregulated at 0h, 12h, and 24h, while there was no significant change at 3h. Specifically, *GmSPS* was upregulated by 0.50-fold, 0.77-fold, and 0.36-fold. *GmA-INV* was significantly upregulated by 0.32-fold, 0.79-fold, and 0.51-fold; *GmN-INV* is significantly upregulated by 0.51-fold, 0.77-fold, and 0.64-fold, respectively. Furthermore, this experiment found that all drought treatments significantly upregulated the relative expression of *GmSUS* in the leaves, with the most pronounced upregulation observed in the W3S treatment. For the W6S treatment, the relative expression of *GmSUS* at 0h showed no significant difference compared to the NC treatment, while at 3h, 12h, and 24h, it was significantly upregulated by 2.77-fold, 2.99-fold, and 0.98-fold, respectively.

**Figure 9:**
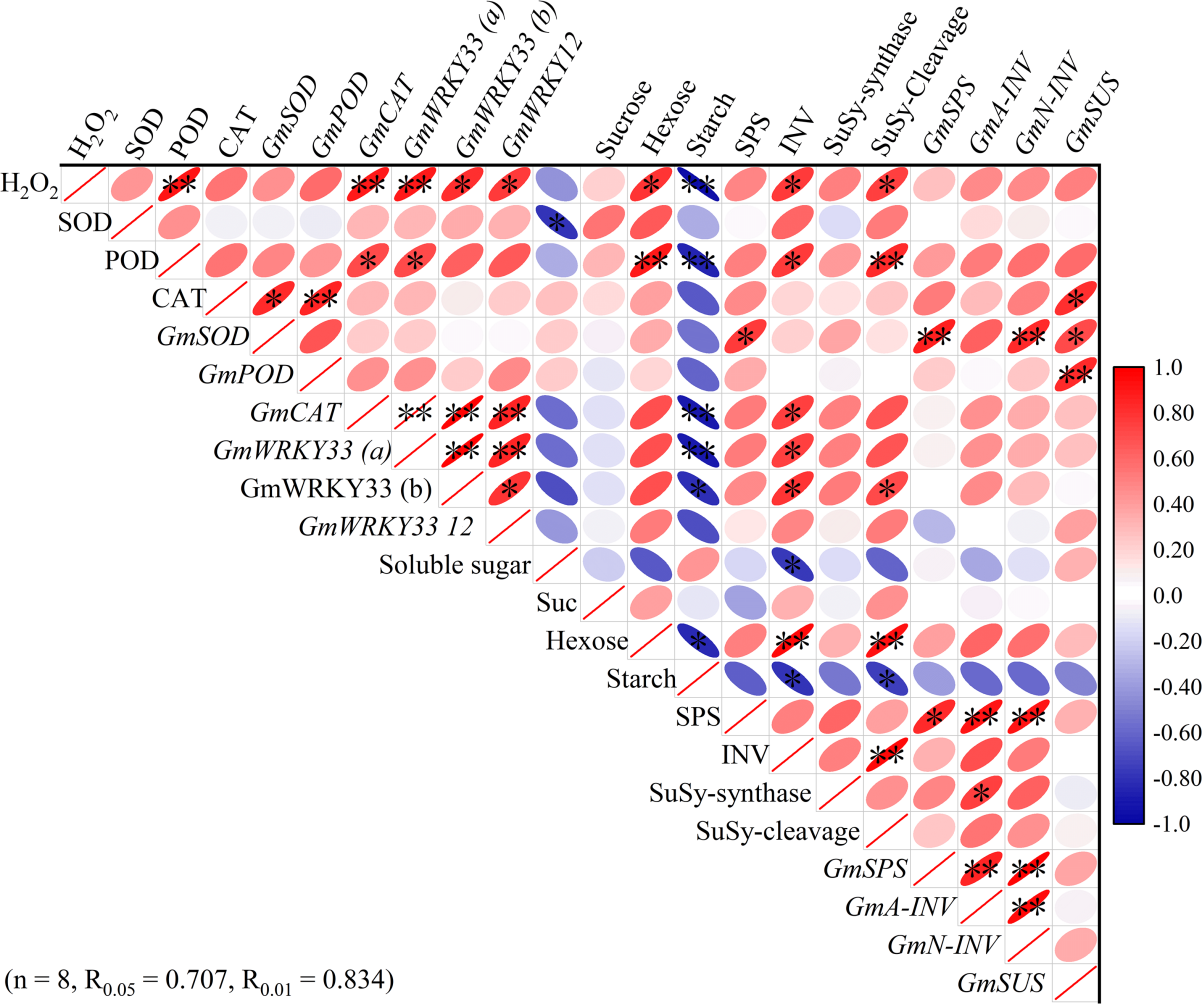
The relationship between H_2_O_2_ homeostasis and WRKY, carbohydrate metabolism, and antioxidant enzyme activity under the W6S treatment.

To elucidate the primary elements affecting the stable production of H_2_O_2_ during W6S treatment, a correlation analysis was additionally performed (**Figure 9**). The results indicated a significant correlation between H_2_O_2_ and *GmWRKY33a*, *GmWRKY33b*, and *GmWRKY12*, suggesting that the establishment of homeostasis under W6S treatment was closely related to the regulation by these three transcription factors. Furthermore, this study found that although there is a highly significant positive correlation between H_2_O_2_ and POD in the leaves, there was no apparent correlation with the regulatory gene *GmPOD*, indicating that POD did not play a major role in the formation of this homeostasis. Notably, we discovered a significant correlation between H_2_O_2_ and the cleavage of sucrose to hexose, as H_2_O_2_ showed a significant positive correlation with hexose, SuSy-cleavage, and INV, which also implied that hexose is required as an osmotic regulator to maintain homeostasis during the establishment process.

## 4 Discussion

### 4.1 Appropriate drought priming promoted the H_2_O_2_ homeostasis formation

Drought resistance in plants can be significantly enhanced through appropriate drought priming prior to exposure to severe drought stress (Sharma et al., 2024). ROS, particularly H_2_O_2_, plays a pivotal role in this enhancement. Previous studies (Shen et al., 2024; Tao et al., 2022) have demonstrated that drought-primed plants accumulated H_2_O_2_ as a signaling molecule, resulting in improved drought tolerance during subsequent severe stress, with the 0.5% PEG6000 treatment proving to be the most effective. In this study, we further investigated the effects of varying durations of 0.5% PEG6000 priming on H_2_O_2_ levels and their relationship with drought resistance in soybean. Our results indicated that seedlings subjected to the W6S treatment exhibited optimal drought resistance, characterized by reduced leaf curling and yellowing compared to other treatments.

Water content is a critical indicator of plant drought response, while relative conductivity and MDA reflect membrane integrity (Zhou et al., 2021). Under drought stress, these parameters are widely used to assess cellular damage and plant resistance (Wang et al., 2018b). In this study, relative water content declined across all treatments compared to the NC group, but the reduction was smallest under W6S. Similarly, the relative conductivity increased with prolonged stress in all treatments, yet the rise was lowest under W6S (**Figure 2**). Although the MDA content of leaves decreased at 3H during the W6S treatment compared to NC, no significant differences were observed during the steady-state phase (**Figure 3**). These results indicate that the W6S condition imposed the least physiological damage on soybean seedlings.

Under stable and adaptive external environmental conditions, the production and scavenging of ROS are dynamically balanced, thereby maintaining H_2_O_2_ homeostasis. However, drought stress disrupts this balance, leading to excessive accumulation of ROS (Samanta et al., 2024). Consequently, the duration of priming is crucial; prolonged priming may induce stress-related damage and disturb H_2_O_2_ homeostasis, while insufficient priming time may not initiate adequate regulatory mechanisms. In this study, although H_2_O_2_ levels under W6S were slightly elevated compared to the NC group, they reached new equilibria at both 3h–12h and 12h–24h (**Figure 3**). This indicates that W6S priming established a stable state of H_2_O_2_, allowing seedlings to first achieve primary homeostasis under mild stress and subsequently develop advanced homeostasis under severe drought conditions.

The regulation of WRKY TFs is closely associated with the dynamics of H_2_O_2_. Based on prior transcriptome data, we analyzed four *WRKY* genes with significantly different altered expression. Among these, *GmWRKY33a*, *GmWRKY12*, and *GmWRKY33b* exhibited marked changes under W3S, W6S, and W12S (**Figure 5**). Their expression patterns reflect stage-specific regulatory roles during the drought response. Notably, stable H_2_O_2_ homeostasis was established only under W6S, which was largely associated with these three genes. During the initial severe stress phase (0h–3h), their expression was strongly upregulated and then gradually normalized as stress signals stabilized, coinciding with the re-establishment of H_2_O_2_ homeostasis. Importantly, a distinct downregulation phase occurred at 12h under W6S, where the expression of *GmWRKY33a/b* and *GmWRKY12* declined to levels near NC (**Figure 5**). This transient suppression likely serves as a negative feedback mechanism to prevent excessive activation of ROS-scavenging enzymes, thereby allowing a moderate, non-damaging H_2_O_2_ elevation (18.4% above NC) to persist as a sustained stress signal. Between 12h and 24h, the plants were in an environment with 5% PEG6000, without any additional stress applied; the regulation of H_2_O_2_ balance became critical. At 24h, the transcription factors in W6S-treated plants rose significantly, mitigating drought damage. Correlation analysis further confirmed that H_2_O_2_ homeostasis under W6S treatment was positively correlated with *GmWRKY33a*, *GmWRKY33b*, and *GmWRKY12*. In light of the above, these results suggest that the dynamic expression of these *WRKY* genes plays a central role in restoring leaf H_2_O_2_ homeostasis and enhancing drought resistance in soybean.

SOD, POD, and CAT are key antioxidant enzymes involved in ROS metabolism (Batool et al., 2022; Zeng et al., 2019). Consistent with the findings on grafted grapevine and rice (Jiao et al., 2023; Wang et al., 2019), the transcription levels and enzyme activities of SOD and POD in soybean leaves of this study increased under drought stress at W3S, W6S, and W12S treatments (**Figure 4**). For CAT, its activity increased while expression of *GmCAT* decreased, indicating that enzyme activity is not solely determined by transcript abundance. In addition, our findings indicated that the steady state achieved in W6S was not primarily driven by antioxidant enzyme activities. Although H_2_O_2_ steady state under W6S was positively correlated with POD activity, the transcription of *GmPOD*, which directly influences POD activity, showed no significant correlation (**Figure 9**). The elevated levels of transcription and enzyme activity of POD and others in response to these treatments might be attributed to the plant’s inherent stress response.

### 4.2 H_2_O_2_ homeostasis formation was closely related to the sucrose cleavage into hexose, promoting osmotic potential

Under drought stress, plants reduce water loss through stomata closure; however, this simultaneously restricts CO_2_ and decreases leaf photosynthetic rates (Zia et al., 2021). To alleviate these effects, plants enhance their water retention capabilities by accumulating osmoregulatory compounds, thereby maintaining normal physiological metabolism under drought stress (Ozturk et al., 2021).

Among these compounds, soluble sugars are considered crucial osmotic regulators that directly affect plant growth and development, playing a vital role in stress adaptation (Afzal et al., 2021). In this study, we observed a significant increase in soluble sugar content under W3S treatment between 0h and 24h, following the transition from mild drought priming to severe drought stress. Similarly, W12S treatment exhibited a notable increase between 0h and 12h, while W6S treatment showed no significant difference compared to the NC treatment, with changes not reaching statistical significance (**Figure 6**). Correlation analysis further indicated that soluble sugar accumulation under W6S treatment was not associated with H_2_O_2_ homeostasis, suggesting the soluble sugars are unlikely to serve as the primary osmotic regulator mediating homeostasis under these treatments.

Hexose, on the other hand, plays a prominent role as an osmotic regulator, can significantly enhance plant drought tolerance (Ozturk et al., 2021). This experiment found that hexose content increased to varying extents under W3S, W6S, and W12S treatment between 0h and 24h, with the lowest increase observed under W6S treatment. Importantly, hexose accumulation under W6S treatment exhibited a significant positive correlation with H_2_O_2_ homeostasis (**Figure 6**), indicating that the formation of H_2_O_2_ homeostasis is closely linked to hexose metabolism. As the primary osmotic regulator, the increased metabolic activity directing hexose degradation provides both substrates and energy necessary for maintaining H_2_O_2_ balance, ensuring stability in materials and energy supply within the leaves and preventing excessive degradation. Conversely, under treatments W3S and W12S, where the duration of severe drought was either too short or too prolonged, soybean plants likely prioritize the allocation of energy and resources toward maintaining metabolic development, which may constrain the establishment of H_2_O_2_ homeostasis. Regarding starch, the starch content significantly decreased between 0h and 24h under treatments W3S, W6S, and W12S, indicating that after the occurrence of adversity, hexose may no longer serve as the primary substrate for conversion to starch.

Carbohydrate metabolism in leaves is primarily regulated by the activities of key enzymes. Drought stress can disrupt the balance by altering the activity of these enzymes (Du et al., 2020). This study found that under W3S, W6S, and W12S treatments, the activities of SPS and SuSy-synthase at 24H were significantly higher than those in the NC treatment, which was accompanied by corresponding upregulation of *GmSPS* and *GmSuSy* (**Figure 7**). Notably, despite the increase observed under W6S treatment, correlation analysis revealed no significant association with H_2_O_2_; thus, this pathway will not be further discussed. In contrast, enzymes involved in INV and SuSy-cleavage, exhibited markedly increased activities under all three treatments at 24h, along with significant upregulation of their regulatory genes. Correlation analysis confirmed that these enzymes play a critical role in influencing H_2_O_2_ homeostasis. Based on these findings, we propose that the sucrose cleavage pathway mediated by INV and SuSy cleavage is a crucial metabolic link affecting leaf H_2_O_2_ homeostasis under drought stress. Under conditions of H_2_O_2_ homeostasis, soybean leaves adopt a highly ‘economical’ strategy to resist drought, maximizing the plant’s drought resistance while maintaining normal physiological functions.

In summary, we systematically evaluated the effects of different drought-priming durations on the establishment of H_2_O_2_ homeostasis in soybean leaves. We clearly demonstrated that a 6h pre-treatment (W6S) could most effectively induce the leaves to form a dynamic balance of H_2_O_2_, thereby enhancing drought resistance. Further analysis indicated that this process relies on the dynamic expression regulation of key transcription factors such as *GmWRKY33a*, *GmWRKY33b*, and *GmWRKY12*. Meanwhile, W6S significantly enhanced the leaf osmotic regulation capacity and antioxidant responses by promoting sucrose cleavage to generate hexoses, thereby facilitating adaptation to drought stress. Our findings not only reveal the signaling and metabolic synergistic mechanisms by which soybeans establish H_2_O_2_ homeostasis under drought stress but also provide a theoretical basis for drought-resistant breeding and adversity management. Meanwhile, it is important to note that our findings are based on a single cultivar, and PEG6000-induced osmotic stress is not entirely equivalent to natural drought conditions in the field. Therefore, as the response of crops to stress is influenced by both genotype and environment (Liu et al., 2026), future soil-based natural drought experiments will be conducted using two cultivars with contrasting drought sensitivities to further validate our results.

## 5 Conclusions

**Figure 10:**
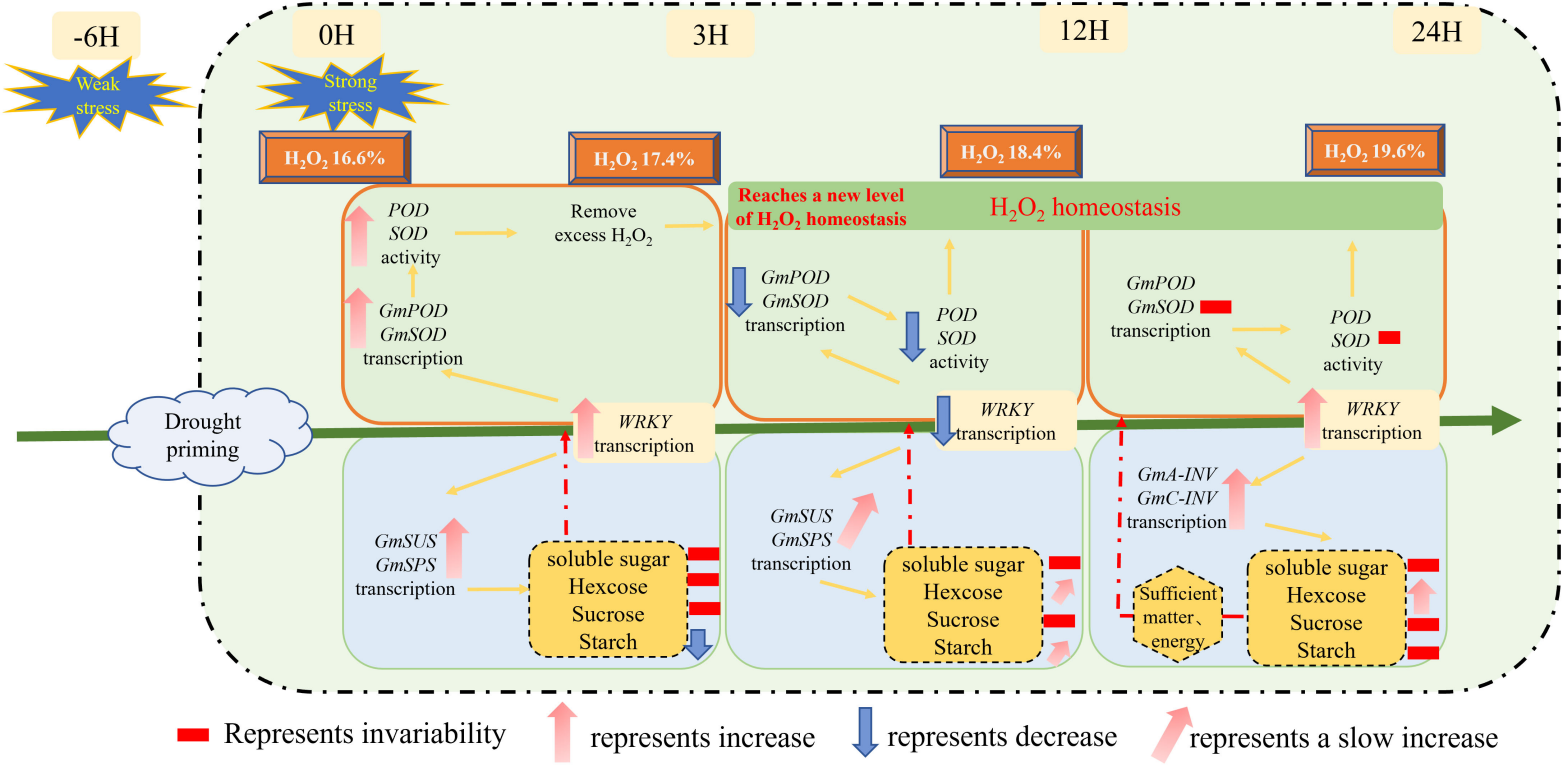
The internal mechanism of H_2_O_2_ homeostasis in soybean under severe drought stress.

Our study presents a model (**Figure 10**) in which soybean leaves form an H_2_O_2_ homeostasis under W6S treatment. H_2_O_2_ homeostasis, under the influence of W6S, exhibited a consistent elevation in content, ranging from a 16.6% to 19.6% increase, throughout 0h to 24h. In the initial stages (0h–3h), the expressions of *WRKY33a/b*, and *WRKY12* were upregulated, accompanied by a surge in peroxidase and catalase gene expressions and activities. Notably, carbohydrate levels remained stable during this time. At 3h–12h, a delicate balance between H_2_O_2_ production and clearance was established. This was evident through the down-regulation of *WRKY33a/b* and *WRKY12*, along with a decrease in peroxidase and catalase activities, while carbohydrate levels continued to remain stable. At 12h–24h, drought conditions further elevated H_2_O_2_ levels, triggering the upregulation of *WRKY33a/b* and *WRKY12*, and activating reactive oxygen species, consequently led to an increase in the expression and activity of sucrose synthase, sucrose phosphate synthase, and invertase genes within the leaves, ultimately resulting in a significant rise in soluble sugar levels. This surge in energy was crucial for maintaining H_2_O_2_ balance and fulfilling the cellular osmotic regulatory requirements.

## Data Availability

The original contributions presented in the study are included in the article/supplementary material. Further inquiries can be directed to the corresponding author.
